# Identification and functional analysis of isopentenyl pyrophosphate isomerase genes in the whiteflies *Bemisia tabaci* (Hemiptera: Aleyrodidae)

**DOI:** 10.1093/jisesa/iead041

**Published:** 2023-06-19

**Authors:** Zhifang Yang, Kui Wang, Shunxiao Liu, Xiang Li, Hongliang Wang, Liuhao Wang, Hongwei Zhang, Hao Yu

**Affiliations:** Department of Natural Resources, Henan Institute of Science and Technology, Xinxiang 453003, Henan Province, China; Department of Natural Resources, Henan Institute of Science and Technology, Xinxiang 453003, Henan Province, China; Department of Natural Resources, Henan Institute of Science and Technology, Xinxiang 453003, Henan Province, China; Department of Plant Protection, College of Agrarian Technology and Natural Resources, Sumy National Agrarian University, Sumy 40021, Ukraine; Department of Natural Resources, Henan Institute of Science and Technology, Xinxiang 453003, Henan Province, China; Department of Natural Resources, Henan Institute of Science and Technology, Xinxiang 453003, Henan Province, China; Department of Natural Resources, Henan Institute of Science and Technology, Xinxiang 453003, Henan Province, China; Department of Natural Resources, Henan Institute of Science and Technology, Xinxiang 453003, Henan Province, China; Department of Natural Resources, Henan Institute of Science and Technology, Xinxiang 453003, Henan Province, China

**Keywords:** *Bemisia tabaci*, isopentenyl, pyrophosphate isomerase, RNA interference, reproduction, juvenile hormone

## Abstract

The juvenile hormone (JH) plays a vital role in the regulation of a number of physiological processes, including development, reproduction, and ovarian maturation. Isopentenyl pyrophosphate isomerase (IPPI) is a key enzyme in the biosynthetic pathway of JH. In this study, we identified an isopentenyl pyrophosphate isomerase protein from *Bemisia tabaci* and named it *BtabIPPI*. The open reading frame (ORF) of *BtabIPPI* is 768 bp and encodes a protein of 255 amino acids that contains a conserved domain of the Nudix family. The temporal and spatial expression profiles showed that *BtabIPPI* was highly expressed in the female adults.RNA interference (RNAi)-mediated silencing of *BtabIPPI* reduced JH titers and the relative expression of vitellogenin receptor (VgR) and JH signaling pathway genes, resulting in a dramatic reduction in fecundity and hatchability. These results indicate that the *BtabIPPI* gene plays an important role in the female fecundity of *B. tabaci*. This study will broaden our understanding of the function of IPPI in regulating insect reproduction and provide a theoretical basis for targeting IPPI for pest control in the future.

## Introduction

Insect growth and reproduction are essentially regulated by 2 major lipophilic hormones, the sesquiterpenoid juvenile hormone (JH) and the steroid called 20-hydroxyecdysone (20E) (Meiselman et al. 2017, Liu et al. 2018). The balance between these 2 hormones determines the outcome of each developmental transition (Dubrovsky 2005). During the growth and development of insects, the steroid hormone 20-hydroxyecdysone (20E) regulates the molting and metamorphosis process through binding with a heterodimer of 2 nuclear receptors, the ecdysone receptor (EcR) and ultraspiracle (USP) (Petryk et al. 2003, Goldsmith et al. 2005). JH synthesized are sesquiterpenoids synthesized by the corpora allata (CA) and play an important role in the prevention of larval metamorphosis and the stimulation of adult reproduction in insects (Bellés et al. 2005, Marchal et al. 2011). While the mechanism of action of 20E is well known, the mechanism of gene regulation by JH is largely unknown.

Terpenoids, also called isoprenoids, are a large and diverse class of organic products (more than 55,000) derived from five-carbon isoprene units (Thulasiram et al. 2007, Vickers et al. 2017). They are widely represented in Eukarya, Archaea, and Bacteria (Zhou 2018). The isoprenoids are synthesized from 2 five-carbon building blocks, isopentenyl pyrophosphate (IPP) and dimethylallyl pyrophosphate (DMAPP). Both kinds of precursors are synthesized through the mevalonate (MVA) and 2-C-methyl-D-erythritol-4-phosphate (MEP) pathways (Diaz et al. 2012, Goodman and Cusson 2012). During the terpenoid biosynthesis process, isopentenyl diphosphate isomerase (IPPI) plays an important role in the isomerization of isopentenyl pyrophosphate (IPP) to dimethylallyl pyrophosphate (DMAPP), IPP and DMAPP are condensed by head to tail reaction to generate geranyl diphosphate (GPP), subsequently, GPP and IPP are condensed to form the JH precursor farnesyl diphosphate (FPP) and geranylgeranyl diphosphate (GGPP) (Anderson et al. 1989, Kuzuyama 2002). Geranylgeranyl diphosphate (GGPP) is used in the biosynthesis of chlorophyll (Zhou et al. 2017). Farnesyl diphosphate (FPP) is the precursor of JH, which is converted into JH via a conserved pathway consisting of isoprenoid-derived metabolites (Vranová et al. 2012).

The whitefly, *Bemisia tabaci* (Hemiptera: Aleyrodidae), is one of the most important agricultural pests worldwide (Jones 2003, Liu et al. 2007). It is a polyphagous insect that feeds on more than 500 plant species, including field crops, vegetables, fruits, and ornamental plants (Brown et al. 1995, Oliveira et al. 2001). They cause extensive yield losses worldwide and become a serious threat to global food security through direct feeding (Wang et al. 2010, Navas-Castillo et al. 2011). Taxonomically, *B. tabaci* is considered as a species complex consisting of many morphologically indistinguishable but genetically distinct and reproductively isolated cryptic species (Barro et al. 2011, Sun et al. 2011). In this cryptic species complex, the Middle East Asia Minor 1 (MEAM1, formerly known as biotype B) and the Mediterranean (MED, formerly known as biotype Q) are highly invasive and have caused considerable economic damage to agriculture worldwide (Hu et al. 2011, Liu et al. 2012). At present, chemical insecticides remain the main approach for management of *B. tabaci*, but the long-term use of chemical insecticides has resulted in many serious problems, including food safety issues, insecticide resistance, and environmental pollution. Thus, it is imperative to explore a green, efficient, and environmentally friendly method to control *B. tabaci* populations. The success of whiteflies largely depends on their strong reproductive abilities. Therefore, understanding the molecular mechanism associated with reproduction is essential for pest management. Previous studies suggest that the isopentenyl-diphosphate isomerase (IPPI) plays an important role in insect growth, development, and fecundity. In *Caenorhabditis elegans*, IPPI is critical for viability, the mutation of isopentenyl-diphosphate isomerase Idi-1 blocked larval growth and development (Yochem et al. 2005). In *Sogatella furcifera* (Horvath) (Hemiptera, Delphacidae), the IPPI gene is involved in the female reproduction process, and knockdown of the *SfIPPI* significantly blocks ovarian development and egg production (Gong et al. 2022b). In addition, IPPI is involved in the JH synthesis process in *Aedes aegypti* (Diaz et al. 2012). However, the role of isopentenyl pyrophosphate isomerase in *B. tabaci* has not been confirmed.

In the study, we cloned an isopentenyl pyrophosphate isomerase gene (*BtabIPPI*) from the genomic database of *B. tabaci* and determined the relative expression level in different developmental stages and tissues by using RT-qPCR. The results showed that *BtabIPPI* was highly expressed in adult females, implying that *BtabIPPI* may be related to the reproduction of *B. tabaci*. Therefore, in the present study, we investigated the function of *BtabIPPI* in females. These results provide a theoretical basis for further experimental studies.

## Materials and Methods

### Insectrearing and Sample Preparation

*Bemisia tabaci* B-biotype (MEAM1) population were reared on cotton plants in separate insect-proof cages in artificial climate chambers at 26°C with a relative humidity 60–70% and 16:8 h light: dark cycle. The purity of the strain was monitored every 3 to 5 generations using the PCR and biomarkers of the cytochrome oxidase I (mtCOI) genes (Chu et al. 2010). Different developmental stages, including eggs, the 4 nymphal stages, and adult females and males, and various tissues, including the head, abdomen, a mixture of the thorax, legs, and wings, were collected from *B. tabaci* MEAM1 populations. Three independent biological replicates were used for each sample. The collected sample was rapidly frozen in liquid nitrogen and stored at −80°C for subsequent RNA isolation and RT-qPCR analysis.

### RNA Isolation and cDNA Synthesis

Total RNA was extracted from individual samples using the TRIzol reagent (Invitrogen, Carlsbad, CA, USA) according to the manufacturer’s instructions. The integrity of the extracted RNA was determined with 1% agarose gel electrophoresis, and RNA concentration and quality were determined using a NanoDrop 2000 spectrophotometer (Thermo Fisher Scientific). All RNA samples were treated with DNase enzyme to eliminate possible contamination of genomic DNA. The first-stand cDNA synthesis was performed using a Prime Script RT Reagent Kit with gDNA Eraser (Takara) according to the manufacturer’s instructions. The synthesized cDNA solution was diluted 10 times with nuclease-free water and used as a template for RT-qPCR reactions.

### Double-stranded RNA Synthesis

Double-strand RNA (dsRNA) was synthesized using the T7 high-yield RNA transcription kit (Vazyme, Nanjing, China) following the manufacturer’s instructions. Based on the nucleotide sequences of *BtabIPPI* and *EGFP*, specific primers containing 6 protective nucleotides and the T7 promoter sequence were designed by Primer Premier 5.0 (Table 1). The enhanced green fluorescent protein gene (*dsEGFP*) was used as a negative control in RNAi experiments. Subsequently, the synthesized dsRNA was further purified via phenol–chloroform precipitation and re-suspended in nuclease-free water. The concentration and quality of dsRNA were evaluated by spectroscopy analysis with NanoDrop 2000 (Thermo Fisher Scientific), and the size of dsRNA was verified by agarose gel electrophoresis. The dsRNA was stored at −80°C until use.

**Table 1.** Primers used for this study

Experiment	Primer	Primer sequence (5ʹ−3ʹ)	Product length
cDNA cloning	*BtabIPPI-F*	CAGCAGGATTCTCGCTCCAT	706 bp
	*BtabIPPI-R*	AGCCCACCATTTCCGTAGTG	
RT-qPCR	*BtabIPPI-F-RT*	CATTGACTCGCTCCTGGCTT	109 bp
	*BtabIPPI-R-RT*	TCTTGCACGGGATCAACCTT	
	*TUB-F*	CACTGTTGTTCCTGGTGGC	151 bp
	*TUB-R*	AGTGGACGAAAGCACGCTTG	
	*BtabVg-F*	ACGTTTGCACCTTTGCCTTC	146 bp
	*BtabVg-R*	GTGGAAAGCGCACTGTTGTT	
	*BtabVgR-F*	GGCTGGCACTGTAGAAGTGT	142 bp
	*BtabVgR-R*	GGCAATGACGAACATCAGGC	
	*BtabKr-h1-F*	AAGCCCTACTCCTGCGAGAT	130 bp
	*BtabKr-h1-R*	GAGTTGAACGTCTCCGAGCA	
	*BtabMet-F*	GTGTCATCGGCAGTGGAGAA	150 bp
	*BtabMet-R*	CAGGAAAGTTGTGTCGGGGT	
dsRNA synthesis	*dsBtabIPPI-F*	TAATACGACTCACTATAGGGGCATTGGGCAGTTCAACCAA	515 bp
	*dsBtabIPPI-R*	TAATACGACTCACTATAGGGCAAACCACGGAGTGACAGGA	
	*dsEGFP-F*	TAATACGACTCACTATAGGGGCCAACCATTGTCACTACTT	486 bp
	*dsEGFP-R*	TAATACGACTCACTATAGGGGGAGTATTTTGTTGATAATGGTCG	

### Sequence Analysis

The cDNA sequence of *BtabIPPI* was identified using TBLASTN from the genome database of *B. tabaci*. The amino acid sequences of IPPI proteins from other insect species were retrieved from the National Center for Biotechnology Information (NCBI). Multiple sequence and identity analyses were carried out using the DNAMAN software (version 5.0; LynnonBioSoft, Quebec, QC, Canada). The theoretical molecular weights (MW) and isoelectric points (pI) of *BtabIPPI* were calculated using the ExPASy web tool (http://ca.expasy.org/tools/pi_tool.html). Exon–intron structure analysis was deduced using GSDS (http://gsds.cbi.pku.edu.cn/). The conserved structural domains of *BtabIPPI* were predicted in the Simple Module Architecture Research Tool (SMART) (http://www.smart.embl-heidelberg.de/) and the Conserved Domains Database (CDD) (http://www.ncbi.nlm.nih.gov/cdd/). Signal peptides were predicted using the Signal P 5.0 server (http://www.cbs.dtu.dk/services/SignalP/). Finally, a phylogenetic tree was constructed by the neighbor-joining method in the MEGA 5.0 software package, and bootstrap analysis was performed with 1,000 replications.

### Reverse Transcription Quantitative Real-time PCR (RT-qPCR)

To determine the expression levels of *BtabIPPI* in different developmental stages and tissues, RT-qPCR was performed with the TB Green Premix Ex TaqTM Kit (Takara Biotechnology) and the 7500 Fast Real-time PCR System (Applied Biosystems). The specific primers are shown in Table 1. The RT-qPCR reaction mixture (20 μL) included 2 μL of diluted cDNA, 10 μL of SYBR Green PCR Master Mix, 0.4 μL of ROX Reference Dye II, 0.8 μL of forward and reverse primers, and 6 μL of nuclease-free water. The cycling conditions were as follows: 95°C for 30 s, followed by 40 cycles of 95°C for 5 s, and 60°C for 34 s. A control without the cDNA template was included in all batches. The housekeeping gene tubulin was used as an internal control (Shen et al. 2010). Each independent experiment was performed in 3 biological replicates, and the relative mRNA level was quantitated using the 2^−∆∆CT^ method (Livak and Schmittgen 2001). The gene-specific primers used in this study are shown in Table 1, and their specificity was checked by the melting curve of the RT-qPCR products.

### RNAi Experiment

To explore the function of *BtabIPPI* in *B. tabaci*, we used RNAi technology to knock down the expression of the target gene by leaf-mediated dsRNA feeding (Luan et al. 2013). Newly-emerged (<2 days) *B. tabaci* MEAM adult were selected as test insects in this study. Briefly, fresh tomato leaflets were cut off from 6-week-old tomato plants and placed in 1.5 ml Eppendorf tubes containing RNase-free water for 2 days for recovery. Then the 2 tomato leaflets were transferred to 2 new 1.5 μL Eppendorf tubes containing 1 ml of solution of dsRNA-IPPI or dsRNA-EGFP (0.5 μg/μL). The open end of the tube was covered with a piece of parafilm. In the end, the 2 Eppendorf tubes with tomato leaflets were transferred into a 50-ml plastic tube covered with a piece of paper towel tightly held with a rubber band. Newly emerged adult whiteflies were released into this silencing system. The solution of dsRNA in the Eppendorf tube was replenished every day. The effectiveness of RNAi was assessed by RT-qPCR at 1, 2, and 3 days post-feeding. To clarify their regulatory relationships between target gene and reproduction-related genes, we determined the transcriptional levels of the JH signal transduction pathway related gene. Six biological replicates were performed in this experiment.

### Bioassay

To confirm the role of the target gene on the fertility of *B. tabaci*, we used RNAi technology to silence the expression of the target gene in newly emerged adult females. Each treated adult female (*dsBtabIPPI* or *dsEGFP*) was placed in a 50-ml plastic tube containing the fresh tomato leaves. Fresh tomato leaves were provided every day. The oviposition rate was estimated as the average number of eggs laid per surviving whitefly. The number of laid eggs was recorded under the microscope daily for 10 days. In addition, the hatching rate was calculated as the number of hatching eggs as a percentage of the total number of eggs laid. The experiment was repeated 6 times. The ovaries of the dsRNA-treated females were dissected in a precooled PBS buffer and photographed with a stereomicroscope (SMZ745, Nikon, Tokyo, Japan).

### JH Titer Determination

To study the effects of *BtabIPPI* silencing on reproduction, the adults were collected from each treatment group (treated with *dsBtabIPPI*) and control group (treated with *dsEGFP*) 6 d after dsRNA feeding. Approximately 200 individuals were pooled as 1 sample. These samples were transferred to Eppendorf tubes with 100 μL of PBS buffer and thoroughly ground using a plastic rod. Next, the samples were centrifuged at 10,000 rpm for 10 min. A volume of 10 μL of the supernatants was dissolved in ELISA buffer. The differences in JH titers between the control and experimental groups were determined by using an insect JH ELISA kit (Shanghai Meilian Biotechnology Co., Ltd.) according to the instructions of the manufacturer. The absorbance (OD) at 450 nm was measured, and the standard curve was plotted using their standards. The experiment was repeated 6 times.

### Statistical Analysis

All statistical analyses were performed using the SPSS software package (SPSS, version 16.0). The relative expression levels of *BtabIPPI* in different developmental stages and tissues were analyzed using one-way analysis of variance followed by Tukey’s honestly significant difference (HSD) multiple comparison test. The significantly differences of the RNAi efficiency, average laid egg numbers, and egg hatching rate were analyzed by Student’s test. The differences were considered statistically significant when *P* < 0.05. Figures were generated using GraphPad Prism 8.0 (GraphPad Software, San Diego, CA, USA).

## Results

### Identification and Sequence Analysis of *BtabIPPI*

**Fig. 1.**
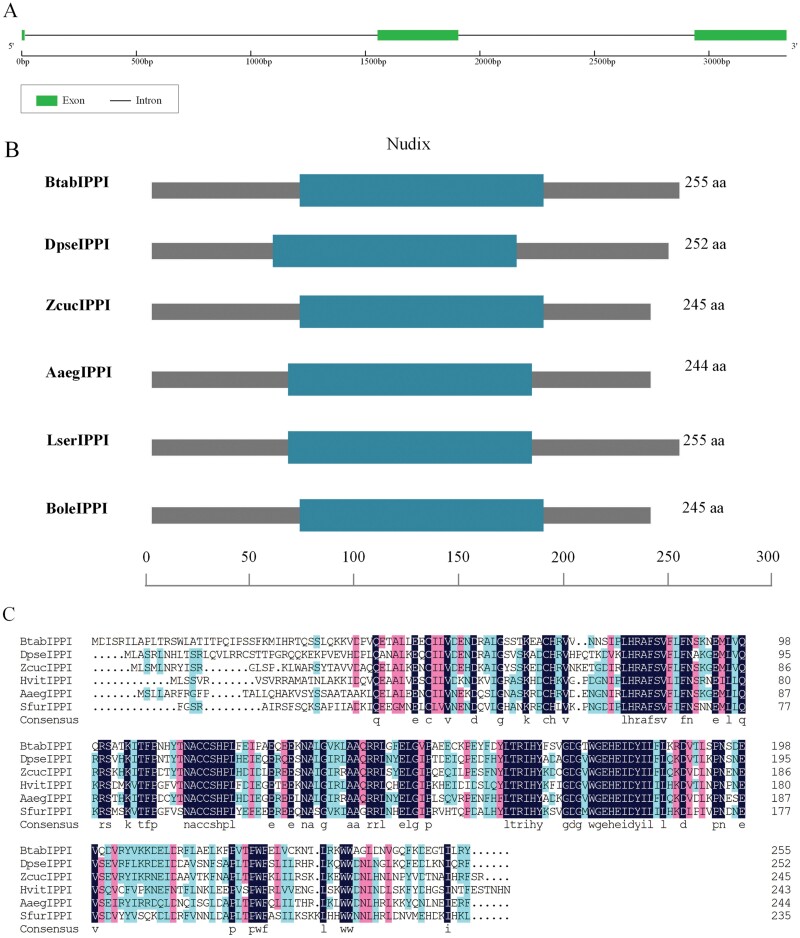
Bioinformatic analysis of *BtabIPPI* in *Bemisia tabaci*. A) Exon/intron structure analysis of the *BtabIPPI* gene. B) A schematic diagram of the structures and deduced proteins of *BtabIPPI*. C) Multiple sequence alignments of the deduced IPPI proteins in insect species.

**Fig. 2.**
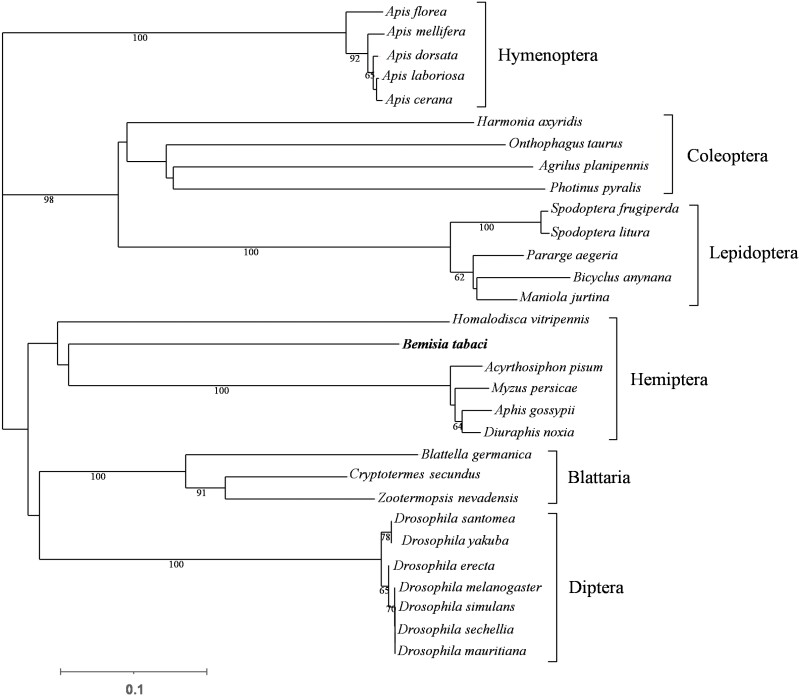
A phylogenetic tree was constructed with the neighbor-joining method of MEGA 7 using the pairwise deletion of indels. Bootstrap support was based on 1,000 resembled data sets. The GenBank accession numbers are listed in Supplementary Table S2.

According to the verification results, the full-length cDNA sequence of *BtabIPPI* (GenBank accession number: XP_018898550.1) in whiteflies contained a 768-bp open reading frame (ORF) that encoded a hypothetical protein sequence of 255 amino acids with a predicted molecular weight (MW) of 29.40 kDa and an isoelectric point (pI) of 6.01. The *BtabIPPI* gene contained 3 exons (Fig. 1A). Bioinformatic analyses revealed that the protein contained a typical conserved domain of Nudix family (PF00293) (Fig. 1B). Multiple sequence alignment of the IPPI gene showed that amino acid residues at some positions are highly conserved in different insect species (Fig. 1C). To examine the phylogenetic relationship between *BtabIPPI* and other insects, a phylogenetic tree was constructed based on the amino acid sequence from 30 species, which included 6 orders. The result showed *BtabIPPI* is conserved in Hemiptera insects (Fig. 2).

### Developmental and Tissues-specific Expression of *BtabIPPI*

**Fig. 3.**
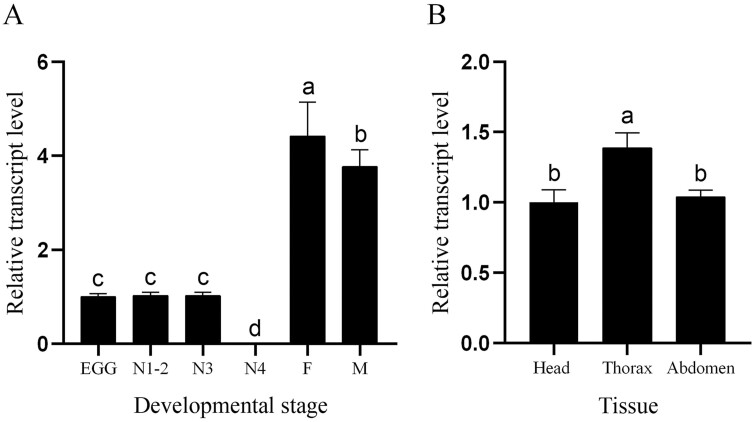
Expression analyses of *BtabIPPI* in developmental stages and different tissues. A) Relative expression levels of *BtabIPPI* in different developmental stages: E = egg, N = nymph stages 1−4 as indicated, F = adult female, and M = adult male. B) Relative expression levels of *BtabIPPI* in different tissues. The standard error for each sample is represented by an error bar, and different letters (a, b, c) above each bar denote significant differences (*P* < 0.05).

The expression patterns of *BtabIPPI* in different tissues and developmental stages were determined by RT-qPCR. The results showed that *BtabIPPI* was expressed in other developmental stages but not in nymphs of the 3rd instar. Among different developmental stages, *BtabIPPI* was highest expressed in adult females (Fig. 3A). Among different tissues types, *BtabIPPI* had the highest relative expression level in thorax, followed by the abdomen, and the lowest expression level was in head (Fig. 3B).

### *BtabIPPI* mRNA Expression Level in *B. tabaci* after dsRNA Feeding

**Fig. 4.**
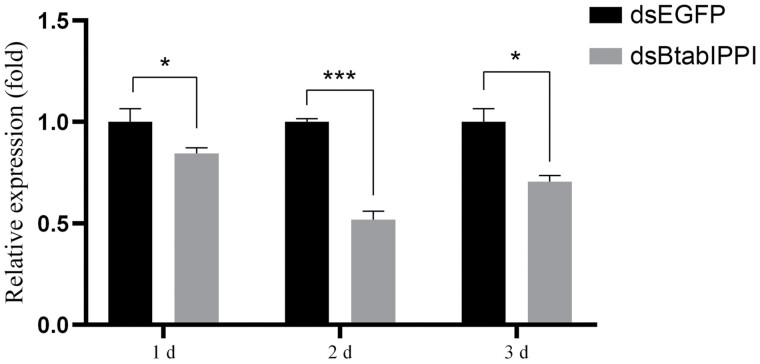
Relative expression levels of *BtabIPPI* in *B. tabaci* after feeding on dsRNA for 1, 2, and 3 days. Data are represented as means ± SE and analyzed by a student’s *t*-test (*P < 0.05; **P < 0.01; ***P < 0.001).

RT-qPCR was performed to detect the mRNA’s relative expression levels after the dsRNA feeding of whiteflies. The results showed that *BtabIPPI* transcript levels were inhibited by 15.60%, 48.18%, and 35.24% in adults treated with *dsBtabIPPI* at 1 day, 2 days, and 3 days, respectively, compared to the control group (*dsEGFP*) (Fig. 4). The results show that RNAi effectively suppressed the expression of target gene in *B. tabaci*.

### Effects of *BtabIPPI* Knockdown on JH Titers and JH-responsive Genes

**Fig. 5.**
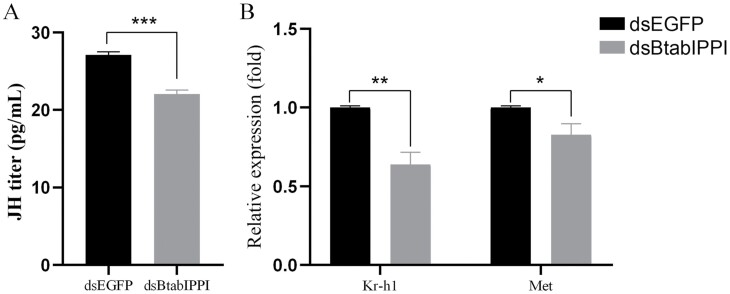
Effects of *BtabIPPI* on JH titles and relative expression levels of JH signaling pathway genes. A) JH titers in female adults after *BtabIPPI* knockdown. B) Effects of *BtabIPPI* knockdown on the relative expression level of JH signaling pathway genes. Every treatment and control were performed in 3 replicates. Data are represented as means ± SE and analyzed by a student’s *t*-test (*P < 0.05; **P < 0.01; ***P < 0.001).

In most female insects, the JH is essential to the reproductive systems of female insects. Therefore, we also recorded fluctuations in hormone titers in adult females in this study. The results showed that RNAi-mediated gene silencing of *BtabIPPI* led to a decreased JH titer, down by 18% compared to the control group (Fig. 5A). This result implies *BtabIPPI* involved in the JH synthesis process. Furthermore, we examined the expression of JH-responsive genes, including *Krüppel homolog 1 (Kr-h1)* and *methoprene-tolerant (Met)*. Results showed that knockdown of *BtabIPPI* significantly reduced the expression of both *Kr-h1* and *Met* (Fig. 5B), confirming the role of *BtabIPPI* in regulating JH signaling.

### Effects of *BtabIPPI* Knock-down on Female Fecundity and Ovarian Development

**Fig. 6.**
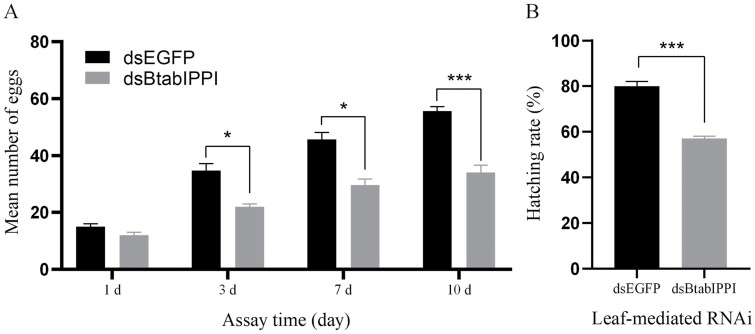
Effects of *BtabIPPI* knockdown on female fecundity. (A) Effects of silencing of *BtabIPPI* on female reproduction. (B) Effects of silencing of *BtabIPPI* on the egg hatching rate of *B. tabaci*. Data are represented as means ± SE and analyzed by a student’s *t*-test (*P< 0.05; **P< 0.01; ***P < 0.001).

**Fig. 7.**
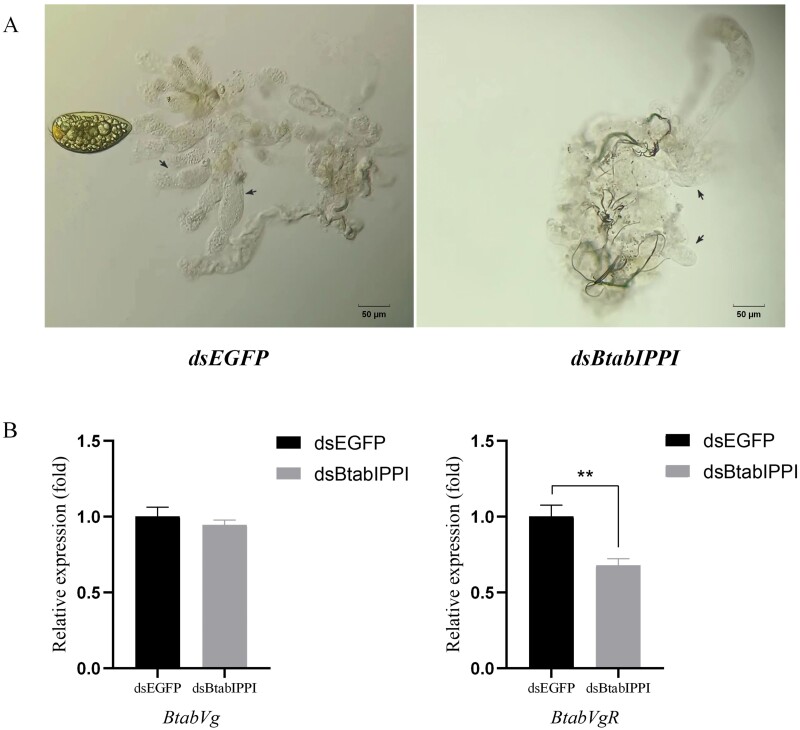
Comparisons of the *B. tabaci* treatment group and control group. A) Effect of *dsBtabIPPI* on *B. tabaci* ovary development. The oocytes of whiteflies are indicated by arrows. The number of mature oocytes in the control group is far greater than that in the treatment group. B) Relative expression of *Vg* and *VgR* after RNAi. Data are represented as means ± SE and analyzed by a student’s *t*-test (*P < 0.05; **P < 0.01; ***P < 0.001).

In this study, we examined the specific function of *BtabIPPI* in female fecundity by comparing egg production and hatching rates between treatment and control groups between the treatment and control groups. The result showed that silencing of *BtabIPPI* reduced female fecundity. After 10 days of *dsBtabIPPI* treatment, the number of females laying eggs was significantly lower than the control group, indicating that *BtabIPPI* plays an important role in female egg laying (Fig. 6A). Furthermore, the knockdown of the *BtabIPPI* gene caused a significant difference in hatch rate as compared to the control group (Fig. 6B). These results indicate that silencing of target gene had caused adverse effects on the female fecundity of *B. tabaci*. To further understand the effect of *BtabIPPI* on the ovarian development of *B. tabaci*, we dissected the ovaries of an adult female at day 6 after dsRNA-feeding. The result showed that silencing of *BtabIPPI* had a significantly negative effect on the female ovaries development. We observed a large number of more fully developed oocytes in the control groups compared to the experiment groups (Fig. 7A). These data suggest that *BtabIPPI* is required for female ovary development.

### Effects of *BtabIPPI* knock-down on the expression Vg and VgR

It has been reported that the vitellogenin (Vg) and vitellogenin receptor (VgR) genes play a key role in insect reproduction and are often used as molecular markers for monitoring the fecundity of insects (Cong et al. 2015, Shang et al. 2018). In order to further understand the mechanism of the effect of *BtabIPPI* gene silencing on female fecundity, we further analyzed the expression of vitellogenin and vitellogenin receptor genes in adult females fed with dsRNA (*dsBtabIPPI* or *dsEGFP*) (Fig. 7B). We found that the expression level of *VgR* was significantly downregulated in treatment groups compared with the expression in control groups. These results indicate that *BtabIPPI* may affect the female reproduction process of *B. tabaci* by regulating the expression of *VgR*. However, the expression levels of *Vg* did not change significantly.

## Discussion

The whitefly, *Bemisia tabaci* (Gennadius) (Hemiptera: Aleyrodidae), is a widely distributed invasive agricultural pest, infesting more than 600 host plant species and causing great loss in agricultural crop production (Perring 2001, Brown and Czosnek 2002). Whiteflies are the most invasive species due to their high fecundity and polyphagous feeding behavior. These factors help them cope with unfavorable environmental conditions. Therefore, understanding insect reproduction behavior and adaptation strategies is crucial to the development of effective management strategies for pest control.

JH is a sesquiterpenoid synthesized by the insect Corpora allata (CA), and it regulates a wide variety of biological events in insects, including embryonic development, metamorphosis, and reproduction (Truman and Riddiford 2002, Jindra et al. 2013). In insects, the absence of JH is a central regulator of reproductive diapause (Li et al. 2022). Isopentenyl pyrophosphate isomerase (IPPI) is a crucial enzyme in the mevalonate pathway in JH synthesis that catalyzes the conversion of isopentenyl pyrophosphate (IPP) to dimethylpropylene pyrophosphate (DMAPP) (Xavier et al. 2005, Diaz et al. 2012). In the present study, we identified and characterized a novel IPPI gene (*BtabIPPI*) from the whiteflies, *B. tabaci*. Bioinformatic and structural analyses revealed that *BtabIPPI* showed high similarities with some membrane protein sequences previously published and contained a typical conserved domain of the Nudix family. Multiple amino acid sequence alignments and evolutionary analysis suggested that the *BtabIPPI* gene was highly conserved across different insect species.

The temporal expression profile of *BtabIPPI* in different developmental stages and in different tissues of *B. tabaci* was detected by RT-qPCR. The results showed that *BtabIPPI* was expressed and fluctuated in all developmental stages, and the highest expression was found in the adult female. Therefore, we speculated that *BtabIPPI* is associated with female fecundity. Many studies have shown that IPPI proteins are involved in the JH pathway and female reproduction. It has been demonstrated that *AaIPPI* plays a critical role in the synthesis of JH in *A. aegypti*, and changing the *AaIPPI* mRNA levels inhibits JH biosynthesis (Diaz et al. 2012). The *SfIPPI* protein is involved in female reproduction, and silencing *SfIPPI* had a negative effect on ovarian development and egg production in *Sogatella furcifera* (Gong et al. 2022a). The above studies have shown that IPPI plays a certain role in female reproduction. In this study, RNAi technology was used to knock down *BtabIPPI* expression and examine its role in female reproduction. The result showed that loss of *BtabIPPI* reduced the JH titers and inhibited ovarian development, oviposition, and egg hatching rate, implying that IPPI might be involved in JH synthesis and play an important role in female fecundity in *B. tabaci*.

In addition, In most insects, vitellogenin (Vg) and the vitellogenin receptor (VgR) play critical roles in the reproductive process of female insects and are typically used as molecular markers of fecundity (Zhai et al. 2015). After the fat body synthesizes vitellogenin protein, it is released into the hemolymph and taken up by developing oocytes through receptor-mediated endocytosis (RME), thereby promoting oocyte development and egg formation. In this process, vitellogenin receptor mediates vitellogenin endocytosis in oviparous insects (Tufail and Takeda 2009, Arrese and Soulages 2010). To better understand the underlying mechanism regarding how *BtabIPPI* silencing disrupts female fecundity, we also examined the expression level of vitellogenin, vitellogenin receptor, and JH signaling pathway genes in females treated with dsRNA (*dsBtabIPPI* or *dsEGFP*). Results showed that successful silencing of the *BtabIPPI* gene significantly inhibited the transcription levels of *VgR*, *Kr-h1*, and *Met*. It seems that the target gene, vitellogenin receptor, and JH signaling pathway genes may be connected or interact in some way. Based on our results, it is reasonable to speculate that *BtabIPPI* affects the number of females laying eggs and egg hatching rates by regulating the transcription of vitellogenin receptors and JH signaling pathway genes. Similar results can be observed in other studies. In *Locusta migratoria*, *LmTDRD5* is involved in female reproduction, knockdown of *LmTDRD5* reduced the transcription level of vitellogenin receptor, thereby impairing ovarian development and oocyte maturation (Deng et al. 2022).

In summary, a novel IPPI gene from *B. tabaci* (named *BtabIPPI*) was identified in the study. The bioinformatic analysis revealed that the *BtabIPPI* protein contains a typical conserved domain of the Nudix family. Knockdown of *BtabIPPI*reduced JH titers and inhibited the number of females laying eggs, egg hatching rates, and the transcription level of vitellogenin receptors and JH signaling pathway genes. The findings suggest that IPPI may restrict female reproduction by regulating the expression of *VgR*, *Kr-h1*, and *Met*. The research will promote a better and deeper understanding of the role of IPPI protein in female fecundity and provide a reference for the control of invasive populations of whiteflies.

## Supplementary Material

iead041_suppl_Supplementary_Material
