# Supplementary material for: Identification and functional analysis of isopentenyl pyrophosphate isomerase genes in the whiteflies Bemisia tabaci (Hemiptera: Aleyrodidae)
Source: J Insect Sci. 2023 Jun 19;23(3):16. doi: 10.1093/jisesa/iead041 (PMC10278561; doi:10.1093/jisesa/iead041)
Supplement: iead041_suppl_Supplementary_Material [file iead041_suppl_supplementary_material.docx]

**Identification and functional analysis of isopentenyl pyrophosphate isomerase genes in the whiteflies (*Bemisia tabaci*)**

**Table S1. The multiple alignment of deduced amino acids of *BtabIPPI* in *Bemisia tabaci***

| **Order** | **Species** | **Gene name** | **GenBank accession no.** | **Sequence length** |
| --- | --- | --- | --- | --- |
| Hemiptera | *Bemisia tabaci* | BtabIPPI | XP_018898550.1 | 255 |
| Diptera | *Drosophila pseudoobscura* | DpseIPPI | XP_001358058.1 | 252 |
|  | *Zeugodacus cucurbitae* | ZcucIPPI | XP_011180923.1 | 245 |
|  | *Aedes aegypti* | AaegIPPI | XP_001657533.1 | 244 |
|  | *Lucilia sericata* | LserIPPI | XP_037807973.1 | 255 |
|  | *Bactrocera oleae* | BoleIPPI | XP_014089794.2 | 245 |

**Table S2. Species and GenBank accession no. for Phylogenetic tree use in the study.**

| **Order** | **Species** | **Gene name** | **GenBank accession no.** |
| --- | --- | --- | --- |
| Diptera | *Drosophila mauritiana* | *DmauIPPI* | XP_033162573.1 |
|  | *Drosophila sechellia* | *DsecIPPI* | XP_002044239.1 |
|  | *Drosophila simulans* | *DsimIPPI* | XP_002102605.1 |
|  | *Drosophila melanogaster* | *DmelIPPI* | NP_001163673.1 |
|  | *Drosophila yakuba* | *DyakIPPI* | XP_002096533.1 |
|  | *Drosophila santomea* | *DsanIPPI* | XP_039490197.1 |
|  | *Drosophila erecta* | *DereIPPI* | XP_001979311.1 |
| Hymenoptera | *Apis florea* | *AfloIPPI* | XP_012345933.1 |
|  | *Apis mellifera* | *AmelIPPI* | XP_003251091.1 |
|  | *Apis cerana* | *AcerIPPI* | XP_016912330.1 |
|  | *Apis dorsata* | *AdorIPPI* | XP_006613763.1 |
|  | *Apis laboriosa* | *AlabIPPI* | XP_043792609.1 |
| Blattaria | *Blattella germanica* | *BgerIPPI* | PSN47596.1 |
|  | *Zootermopsis nevadensis* | *ZnevIPPI* | XP_021940348.1 |
|  | *Cryptotermes secundus* | *CsecIPPI* | XP_023725800.1 |
| Lepidoptera | *Pararge aegeria* | *PaegIPPI* | XP_039758054.1 |
|  | *Maniola jurtina* | *MjurIPPI* | XP_045776879.1 |
|  | *Bicyclus anynana* | *BanyIPPI* | XP_023934716.1 |
|  | *Spodoptera litura* | *SlitIPPI* | XP_022818024.1 |
|  | *Spodoptera frugiperda* | *SfruIPPI* | XP_035449612.1 |
| Coleoptera | *Photinus pyralis* | *PpyrIPPI* | XP_031346170.1 |
|  | *Harmonia axyridis* | *HaxyIPPI* | XP_045461280.1 |
|  | *Agrilus planipennis* | *AplanIPPI* | XP_018335001.1 |
|  | *Onthophagus taurus* | *OtauIPPI* | XP_022909718.1 |
| Hemiptera | *Bemisia tabaci* | *BtabIPPI* | XP_018898550.1 |
|  | *Diuraphis noxia* | *DnoxIPPI* | XP_015375276.1 |
|  | *Myzus persicae* | *MperIPPI* | XP_022170706.1 |
|  | *Aphis gossypii* | *AgosIPPI* | XP_027841489.1 |
|  | *Acyrthosiphon pisum* | *ApisIPPI* | NP_001152834.1 |
|  | *Homalodisca vitripennis* | *HvitIPPI* | XP_046658798.1 |

**Figure S1. Gene silencing system.**


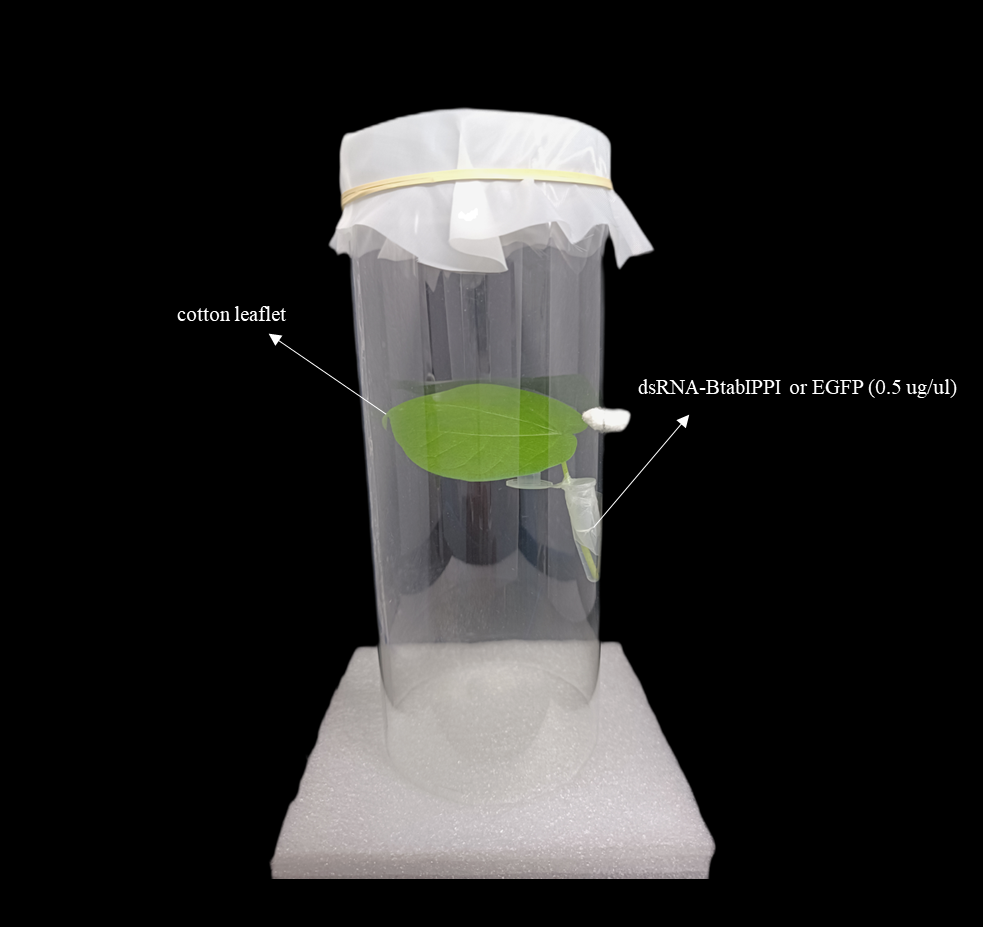


**Target gene silencing through leaf-mediated dsRNA feeding. The cotton leaflets placed in 1.5 ml Eppendorf tubes containing 1ml of solution of dsRNA-*BtabIPPI* or dsRNA-*EGFP* (0.5 ug/ul). The open end of the tube was covered with a piece of parafilm. Newly emerged adults (< 2 days old) of *B. tabaci* were then released onto the leaf through a hole in the plastic container. The dsRNA solution in the Eppendorf tube was replenished every day.**
